# Predictors of risky alcohol consumption in schoolchildren and their implications for preventing alcohol-related harm

**DOI:** 10.1186/1747-597X-2-15

**Published:** 2007-05-10

**Authors:** Mark A Bellis, Karen Hughes, Michela Morleo, Karen Tocque, Sara Hughes, Tony Allen, Dominic Harrison, Eduardo Fe-Rodriguez

**Affiliations:** 1 Centre for Public Health, Liverpool John Moores University, Castle House, North Street, Liverpool, L3 2AY, UK; 2 Trading Standards North West Under Age Sales Strategy Group, North House, 130 Rochdale Road, Oldham, OL1 2JA, UK; 3 NHS North West/Department of Health, Government Office North West, City Tower, Piccadilly Plaza, Manchester, M1 4BE, UK

## Abstract

**Background:**

While alcohol-related health and social problems amongst youths are increasing internationally, both consumption and associated harms are particularly high in British youth. Youth drinking patterns, including bingeing, frequent drinking and drinking in public spaces, are associated with increased risks of acute (e.g. violence) and long-term (e.g. alcohol-dependence) health problems. Here we examine economic, behavioural and demographic factors that predict these risky drinking behaviours among 15–16 year old schoolchildren who consume alcohol. A cross-sectional survey was conducted among schoolchildren in North West England (n = 10,271) using an anonymous questionnaire delivered in school settings. Analysis utilised logistic regression to identify independent predictors of risky drinking behaviour.

**Results:**

Of all respondents, 87.9% drank alcohol. Of drinkers, 38.0% usually binged when drinking, 24.4% were frequent drinkers and 49.8% drank in public spaces. Binge, frequent and public drinking were strongly related to expendable income and to individuals buying their own alcohol. Obtaining alcohol from friends, older siblings and adults outside shops were also predictors of risky drinking amongst drinkers. However, being bought alcohol by parents was associated with both lower bingeing and drinking in public places. Membership of youth groups/teams was in general protective despite some association with bingeing.

**Conclusion:**

Although previous studies have examined predictors of risky drinking, our analyses of access to alcohol and youth income have highlighted eradicating underage alcohol sales and increased understanding of children's spending as key considerations in reducing risky alcohol use. Parental provision of alcohol to children in a family environment may also be important in establishing child-parent dialogues on alcohol and moderating youth consumption. However, this will require supporting parents to ensure they develop only moderate drinking behaviours in their children and only when appropriate.

## Background

In recent years increasing levels of hazardous and harmful alcohol use, along with their negative health consequences, have received international attention as issues requiring immediate action. Thus, the World Health Organization (WHO) has identified excessive alcohol use as one of the most important risks to health [1] and the World Health Assembly (Resolution WHA58.26) has requested Member States to implement effective interventions to tackle alcohol misuse [2,3]. Within the WHO European Region, where per capita alcohol consumption and related disease burden are double global averages [4,5], two successive alcohol action plans (covering 1992 to 2005) have been followed by a Regional Framework providing strategic guidance to member states on the development and implementation of alcohol policy [6]. Further strategic support in the European Union (EU) has been provided through the EU strategy to assist member states in reducing alcohol-related harm [7]. In both, particular attention is focused on the increasing levels of alcohol intoxication and the disproportionate burden of alcohol-related harms in European youth.

Among schoolchildren (aged 15–16) in many European countries, hazardous and harmful drinking patterns have escalated with increases in both binge drinking (here drinking five or more alcoholic drinks in one drinking occasion) (e.g. UK, Norway, Czech Republic) and drunkenness (having ever experienced more than 20 drunken episodes) (e.g. Ireland, Estonia, Slovenia) [8]. In the long term continued heavy alcohol consumption, including frequent bingeing, is linked to pathologies including liver disease, cancers and cardiovascular disease [9-11]. However, binge drinking by youths is also associated with acute problems including alcohol-related violence and other anti-social behaviours [12,13]. Furthermore, it contributes to unprotected sex, drug consumption and educational problems including truancy [13-15].

In the UK, levels of both alcohol-related health burdens (e.g. liver disease) and crime are rising [16,17]. Approximately 7.1 million residents in England are now thought to be hazardous or harmful users of alcohol [18] with around 1.1 million being alcohol dependent [19]. Further, UK youth now have among the highest levels of alcohol consumption and binge drinking in the European Union (e.g. prevalence of binge drinking in the past 30 days among girls aged 15–16: UK 56%, Belgium 44%, France 23%) [8], and experience drunkenness at an earlier age than most of their European counterparts [20]. Associated problems including anti-social behaviour [13] and unintended pregnancy [21] are also high [22,23]. Nationally, alcohol-related problems are estimated to cost approximately £20 billion per annum [24].

The reasons why alcohol creates a disproportionate health and social burden in some cultures (e.g. UK) are likely to be multi-faceted. While debate continues over whether any alcohol consumption by youths can be beneficial [25], studies suggest that drinking behaviours early in life can not only be of immediate detriment to health but may also relate to adult alcohol consumption patterns and their related health consequences [26-29]. In particular, binge drinking and unsupervised underage drinking in public places can result in negative health consequences (e.g. accidents) and are strongly linked to anti-social behaviour [30,31]. However, where alcohol is typically integrated into family life (e.g. in Mediterranean countries such as France and Italy) [25], children display lower levels of binge drinking and alcohol-related anti-social behaviour than their Northern counterparts (e.g. UK and Scandinavian countries) [8]. The consequences of frequent drinking by youths are less clear yet several studies have associated frequent youth drinking with increased health risks including involvement in physical fights [31,32].

Previous research has identified a range of factors associated with risky drinking behaviours in youths. These include those related to: the individual (e.g. sensation seeking, anxiety, positive alcohol expectancies [33]), their relationships (e.g. parental and peer drinking behaviour [34,35]), and the drinking context (e.g. cultural norms, alcohol availability [35]). Here however, we use a school-based (aged 15–16) sample of 10,271 children and focus specifically on those that drink (n = 9,029). We examine relationships between risk-related drinking behaviours and alcohol consumers' demographics, personal economics and access to alcohol and leisure activities. Finally, we explore how an understanding of such relationships can be used to reduce alcohol-related harm.

## Methods

Within England, the North West Region (population 6.8 million) [36] has the highest number and greatest prevalence of hazardous/harmful drinkers (1.25 million) of all English Regions [19]. Previous work within the Region has already identified both substantial consumption of alcohol by young people and frequent illegal alcohol sales to those under 18 (UK minimum age of purchase is 18) [37-39]. To provide more intelligence on both issues, Trading Standards North West (the organisation responsible for enforcing legislation and regulations governing the sale of goods and services, including alcohol, in the North West of England) undertook a survey of 14 to 17 year old schoolchildren in the North West Region. Questions were anonymous and explored: basic demographics of respondents; drinking frequencies (over the past six months), types and quantities of alcohol consumed (drinks were counted in the number of bottles, cans, glasses and pints consumed to accommodate existing definitions of youth bingeing [8]); sources of alcohol; income available to youths; and personal views on alcohol consumption. The questionnaire consisted almost entirely of closed questions with the exception of one requesting suggestions for additional facilities for young people. The questionnaire was made freely available though local Trading Standards offices (n = 22 covering the North West Region of England) to schools located within their administrative areas. In schools agreeing to participate, questionnaires were distributed by Trading Standards Officers to school staff (teachers and assistants) who then delivered the questionnaire to students within normal school lessons. Research methods and materials complied with the Declaration of Helsinki. Compliance levels were not recorded in each class as the sample was not intended to be representative but was opportunistic, with analyses focusing on relationships between variables recorded by individual participants. Further, while demographics such as ethnicity were included, only broad categories were used in order to obtain meaningful sample sizes in each group.

From 147 North West secondary schools (in 21 local authorities) that participated in the survey, a total of 12,883 questionnaires were returned during the sampling period (from 1^st ^February to 31^st ^March 2005). From these, 43 were excluded immediately due to completion by 18 year olds. In order to assign an approximate level of deprivation to each school, Index of Multiple Deprivation 2004 (IMD) [40] was calculated based on the Middle Super Output Area (MSOA) in which each school was located. For the North West, MSOA are geographical areas with an average total population of 7,380 (500 for those aged 15–19). Based on the IMDs of all MSOAs across the North West, each MSOA was allocated to a regional quintile of deprivation and students were allocated to quintiles based on the MSOA of their school. At this stage any schools that were not identified as local state schools (i.e. tax funded and recruiting in general from the local population) were removed from the analysis (n = 15 schools), leaving 132 general secondary schools out of a total of 474 such schools in the North West [41]. In addition, as only 1% of pupils were 17 years old and 2% 14 years old, analyses were limited to 15 to 16 year olds where adequate numbers were available. The final sample for analysis was 10,271 respondents.

Initial analyses (Table 1) examined factors predicting whether individuals drink alcohol at all (here defined as drinking at least once every six months). However, most analyses (Tables 2 and 3) focused on identifying predictors of whether those that consume alcohol: usually have five or more alcoholic drinks per session (here, binge drinking); usually drink at least two or more days each week (here, frequent drinking); and drink in public settings (including drinking in streets, parks, pubs and clubs and excluding drinking at home, in friends' houses and on special occasions with family and friends). From these three risk factors, two composite variables were also calculated: "any drinking risk" (having any one of the three risk factors); and "all drinking risks" (having all three risk factors). Initial chi square statistics were calculated in SPSS [42]. However, to account for confounding relationships between variables and potential underestimation of variance associated with cluster sampling [43,44] STATA was utilised for logistic regression with appropriate cluster sample corrections applied [45].

**Table 1:** General sample characteristics and comparisons between those that do and do not consume alcohol

			**Drink alcohol**					
	**All**		**No**		**Yes**		**Statistic**	
	n	%	n	%	n	%	Chi Square	P
**Sex**								
Female	5366	52.2	515	9.6	4826	90.4		
Male	4905	47.8	676	13.9	4203	86.1	43.96	< 0.001
**Age**								
15	4834	47.1	593	12.3	4217	87.7		
16	5437	52.9	598	11.1	4812	88.9	4.02	< 0.05
**North West deprivation quintile**								
(Least deprived) 1	2560	24.9	172	6.7	2384	93.3		
2	2324	22.6	338	14.6	1975	85.4		
3	1472	14.3	148	10.1	1317	89.9		
4	2354	22.9	317	13.6	2019	86.4		
(Most deprived) 5	1561	15.2	216	13.9	1334	86.1	99.48	< 0.001
**Ethnicity**								
White	8606	87.5	507	5.9	8077	94.1		
Black/Black British	151	1.5	26	17.7	121	82.3		
Chinese/Chinese British	84	0.9	22	26.2	62	73.8		
Asian/Asian British	701	7.1	546	78.9	146	21.1		
Mixed Race	239	2.4	17	7.1	221	92.9		
Other	49	0.5	19	39.6	29	60.4	3389.43	< 0.001
**Spending money (£/week)**								
< = 10	3633	38.7	546	15.1	3070	84.9		
11 to 20	3047	32.4	307	10.1	2733	89.9		
21 to 30	1247	13.3	85	6.8	1160	93.2		
> 30	1470	15.6	111	7.6	1354	92.4	102.27	< 0.001
**Ever buy alcohol**								
No	6450	65.2	1191	18.5	5259	81.5		
Yes	3449	34.8	0	0.0	3449	100	723.97	< 0.001
**Member of youth club, group or team**								
No	6411	63.4	774	12.1	5608	87.9		
Yes	3706	36.3	397	10.7	3297	89.3	4.34	< 0.05

Analyses utilise chi squared. Samples sizes vary slightly between analyses due to some respondents not completing all questions.

**Table 2:** Relationships between drinking behaviours and consumers' demographics, methods of accessing alcohol and income

		**Drinking behaviours**^**1**^														
		**Binge drink**			**Frequently drink**			**Drink in public setting**			**Any drinking Risks**			**All Drinking Risks**		
		n (Yes)	%	P	n (Yes)	%	P	n (Yes)	%	P	n (Yes)	%	P	n (Yes)	%	P
**Sex**	Female	1672	34.6		1002	21.2		2339	50.4		3169	65.7		348	7.7	
	Male	1755	41.8	< 0.001	1146	28.1	< 0.001	1998	49.0	0.191	2877	68.5	< 0.01	462	11.7	< 0.001
**Age**	15	1513	35.9		892	21.8		1904	46.9		2715	64.4		295	7.5	
	16	1914	39.8	< 0.001	1256	26.7	< 0.001	2433	52.2	< 0.001	3331	69.2	< 0.001	515	11.3	< 0.001
**Deprivation quintile**	(Least deprived) 1	873	36.6		539	23.0		1047	45.1		1535	64.4		194	8.5	
	2	775	39.2		448	23.3		925	48.6		1339	67.8		154	8.3	
	3	515	39.1		325	25.1		639	50.4		879	66.7		128	10.3	
	4	767	38.0		509	26.0		1046	53.6		1383	68.5		201	10.6	
	(Most deprived) 5	497	37.3	0.38	327	25.3	0.117	680	53.4	< 0.001	910	68.2	< 0.05	133	10.8	< 0.05
**Ethnicity**	White	3121	38.6		1877	23.8		3869	49.5		5409	67.0		724	9.5	
	Black/Black British	25	20.7		19	16.4		51	44.0		63	52.1		2	1.8	
	Chinese/Chinese British	15	24.2		14	23.7		21	35.6		31	50.0		5	8.9	
	Asian/Asian British	44	30.1		43	30.1		64	49.2		91	62.3		10	7.8	
	Mixed Race	83	37.6		58	26.7		133	61.9		168	76.0		26	12.3	
	Other	8	27.6	< 0.001	8	30.8	0.143	17	63.0	< 0.005	22	75.9	< 0.001	2	8.3	0.069
**Spending money (£/week)**	< = 10	951	31.0		454	15.2		1110	37.9		1716	55.9		117	4.1	
	11 to 20	1152	42.2		670	25.0		1414	53.4		1927	70.5		280	10.8	
	21 to 30	522	45.0		372	32.5		661	58.5		880	75.9		162	14.5	
	> 30	628	46.4	< 0.001	494	37.1	< 0.001	861	64.8	< 0.001	1105	81.6	< 0.001	212	16.2	< 0.001
**Ever buy alcohol**	No	1689	32.1		831	16.3		1739	33.1		2958	56.2		221	4.3	
	Yes	1737	50.4	< 0.001	1315	38.8	< 0.001	2596	75.3	< 0.001	3086	89.5	< 0.001	588	17.3	< 0.001
**Member of youth club, group or team**	No	2070	36.9		1426	26.1		2769	51.0		3796	67.7		530	10.0	
	Yes	1317	39.9	< 0.005	686	21.4	< 0.001	1512	47.8	< 0.005	2169	65.8	0.065	268	8.7	< 0.05

**Others buying youths alcohol**																
Parents	No	1948	41.3		1251	27.1		2833	64.0		3550	75.3		522	12.1	
	Yes	1479	34.5	< 0.001	897	21.5	< 0.001	1504	35.0	< 0.001	2496	58.2	< 0.001	288	6.9	< 0.001
Older siblings	No	2908	37.0		1803	23.5		3695	48.8		5165	65.7		675	9.2	
	Yes	519	45.2	< 0.001	345	30.7	< 0.001	642	56.0	< 0.001	881	76.8	< 0.001	135	12.0	< 0.005
Friends (over 18)	No	2076	34.3		1258	21.4		2464	42.8		3612	59.8		454	8.1	
	Yes	1351	45.6	< 0.001	890	30.6	< 0.001	1873	63.2	< 0.001	2434	82.1	< 0.001	356	12.3	< 0.001
Friends (under 18)	No	2662	36.7		1656	23.5		3241	46.6		4647	64.1		605	8.9	
	Yes	765	43.5	< 0.001	492	28.4	< 0.001	1096	62.4	< 0.001	1399	79.6	< 0.001	205	11.8	< 0.001
Adult outside shops	No	2998	37.5		1780	22.8		3515	45.6		5123	64.0		663	8.8	
	Yes	429	42.6	< 0.005	368	37.2	< 0.001	822	81.7	< 0.001	923	91.7	< 0.001	147	14.9	< 0.001
Take/steal from parents	No	3157	37.9		1916	23.6		3959	49.3		5528	66.4		728	9.3	
	Yes	270	39.4	0.44	232	34.8	< 0.001	378	55.2	< 0.005	518	75.6	< 0.001	82	12.3	< 0.05

1 Drinking behaviours are defined as binge drink (usually having five or more alcoholic drinks per session); frequently drink (usually drinking at least two or more days each week) and drink in public settings (including drinking in streets, parks, pubs and clubs and excluding drinking at home, in friends' houses and on special occasions with family and friends). "Any drinking risks" and "all drinking risks" are composite variables calculated as having any one of the three individual risk factors and having all three risk factors respectively. All analyses utilise chi squared.

**Table 3:** Logistic regression analysis of relationships between alcohol consumers' demographics, methods of accessing alcohol, income and drinking behaviours

		**Drinking behaviour**^**1**^									
		**Binge drink**		**Frequently drink**		**Drink in public settings**		**Any risk**		**All risks**	
		AOR (95%CI)	P	AOR (95%CI)	P	AOR (95%CI)	P	AOR (95%CI)	P	AOR (95%CI)	P
	Sample Size	7704		7536		7704		7704		7536	

**Sex**	Female	Ref		Ref		Ref		Ref		Ref	
	Male	1.35 (1.20–1.53)	< 0.001	1.46 (1.27–1.68)	< 0.001	0.88 (0.77–1.00)	0.055	1.11 (0.96–1.29)	0.153	1.61 (1.31–1.98)	< 0.001
**Age**	15	Ref		Ref		Ref		Ref		Ref	
	16	1.06 (0.96–1.18)	0.256	1.11 (0.98–1.25)	0.091	1.12 (0.99–1.26)	0.076	1.09 (0.97–1.22)	0.162	1.35 (1.12–1.63)	< 0.005
**Deprivation quintile**	(Least deprived) 1	Ref		Ref		Ref		Ref		Ref	
	2	1.16 (0.95–1.42)	0.151	0.99 (0.80–1.23)	0.939	1.14 (0.92–1.40)	0.236	1.27 (1.01–1.58)	< 0.05	0.95 (0.75–1.20)	0.650
	3	1.21 (0.97–1.51)	0.092	1.15 (0.92–1.43)	0.225	1.26 (1.01–1.57)	< 0.05	1.24 (1.00–1.53)	< 0.05	1.19 (0.89–1.59)	0.232
	4	1.09 (0.86–1.39)	0.456	1.17 (0.96–1.42)	0.113	1.33 (1.06–1.69)	< 0.05	1.23 (1.01–1.51)	< 0.05	1.21 (0.96–1.54)	0.111
	(Most deprived) 5	1.07 (0.78–1.47)	0.652	1.15 (0.92–1.44)	0.227	1.17 (0.93–1.48)	0.178	1.15 (0.89–1.49)	0.291	1.23 (0.89–1.69)	0.207
**Ethnicity**	White	Ref		Ref		Ref		Ref		Ref	
	Black/Black British	0.66 (0.33–1.29)	0.221	0.50 (0.20–1.22)	0.128	0.93 (0.43–2.01)	0.850	0.58 (0.28–1.19)	0.134	0.31 (0.06–1.45)	0.134
	Chinese/Chinese British	0.81 (0.37–1.74)	0.583	0.83 (0.39–1.78)	0.636	0.91 (0.39–2.13)	0.820	0.72 (0.31–1.69)	0.453	1.45 (0.42–4.99)	0.551
	Asian/Asian British	1.70 (1.01–2.86)	< 0.05	0.96 (0.60–1.54)	0.859	1.62 (0.90–2.94)	0.110	1.62 (0.94–2.79)	0.079	1.99 (0.88–4.51)	0.097
	Mixed Race	1.43 (0.77–2.64)	0.251	1.04 (0.59–1.82)	0.901	2.02 (0.98–4.13)	0.055	1.97 (0.96–4.04)	0.064	2.35 (0.96–5.73)	0.061
	Other	0.80 (0.29–2.20)	0.668	0.76 (0.29–2.20)	0.662	1.54 (0.45–5.29)	0.493	1.68 (0.63–4.50)	0.300	0.95 (0.16–5.72)	0.957
**Spending money (£/week)**	< = 10	Ref		Ref		Ref		Ref		Ref	
	11 to 20	1.38 (1.21–1.58)	< 0.001	1.57 (1.35–1.83)	< 0.001	1.51 (1.30–1.76)	< 0.001	1.47 (1.28–1.68)	< 0.001	2.36 (1.79–3.10)	< 0.001
	21 to 30	1.50 (1.28–1.76)	< 0.001	2.18 (1.81–2.36)	< 0.001	1.72 (1.42–2.08)	< 0.001	1.90 (1.57–2.30)	< 0.001	3.04 (2.26–4.09)	< 0.001
	> 30	1.50 (1.27–1.78)	< 0.001	2.37 (1.98–2.84)	< 0.001	2.01 (1.69–2.39)	< 0.001	2.25 (1.82–2.79)	< 0.001	3.11 (2.37–4.07)	< 0.001
**Ever buy alcohol**	No	Ref		Ref		Ref		Ref		Ref	
	Yes	1.85 (1.64–2.09)	< 0.001	2.88 (2.53–3.27)	< 0.001	5.72 (4.96–6.60)	< 0.001	5.92 (5.02–6.97)	< 0.001	3.53 (2.90–4.30)	< 0.001
**Member of youth club, group or team**	No	Ref		Ref		Ref		Ref		Ref	
	Yes	1.09 (0.98–1.21)	0.128	0.71 (0.63–0.81)	< 0.001	0.90 (0.80–1.00)	0.052	0.92 (0.81–1.04)	0.192	0.77 (0.66–0.90)	< 0.005

**Others buying youths alcohol**											
Parents	No	Ref		Ref		Ref		Ref		Ref	
	Yes	0.82 (0.73–0.92)	< 0.005	1.00 (0.88–1.15)	0.954	0.51 (0.45–0.57)	< 0.001	0.67 (0.59–0.76)	< 0.001	0.79 (0.65–0.96)	< 0.05
Older siblings	No	Ref		Ref		Ref		Ref		Ref	
	Yes	1.31 (1.12–1.52)	< 0.005	1.20 (1.02–1.43)	< 0.05	1.31 (1.08–1.58)	< 0.01	1.49 (1.22–1.81)	< 0.001	1.20 (0.96–1.51)	0.112
Friends (over 18)	No	Ref		Ref		Ref		Ref		Ref	
	Yes	1.40 (1.24–1.58)	< 0.001	1.43 (1.26–1.63)	< 0.001	2.13 (1.86–2.45)	< 0.001	2.42 (2.07–2.83)	< 0.001	1.46 (1.20–1.76)	< 0.001
Friends (under 18)	No	Ref		Ref		Ref		Ref		Ref	
	Yes	1.19 (1.05–1.35)	< 0.01	1.09 (0.94–1.28)	0.251	1.74 (1.47–2.07)	< 0.001	1.78 (1.46–2.17)	< 0.001	1.28 (1.03–1.58)	< 0.05
Adult outside shops	No	Ref		Ref		Ref		Ref		Ref	
	Yes	0.97 (0.82–1.15)	0.739	1.58 (1.32–1.89)	< 0.001	4.84 (3.85–6.09)	< 0.001	4.71 (3.53–6.30)	< 0.001	1.37 (1.06–1.77)	< 0.05
Take/steal from parents	No	Ref		Ref		Ref		Ref		Ref	
	Yes	0.89 (0.75–1.06)	0.175	1.33 (1.09–1.63)	< 0.01	1.09 (0.85–1.40)	0.497	1.19 (0.93–1.51)	0.163	1.03 (0.74–1.45)	0.848

1 Drinking behaviours are defined as binge drink (usually having five or more alcoholic drinks per session); frequently drink (usually drinking at least two or more days each week) and drink in public settings (including drinking in streets, parks, pubs and clubs and excluding drinking at home, in friends' houses and on special occasions with family and friends). "Any drinking risk" and "all drinking risks" are composite variables calculated as having any one of the three individual risk factors and having all three risk factors respectively. Logistic regression was undertaken in STATA and utilised appropriate corrections for cluster sampling (see methods). Sample sizes vary as individuals not answering all questions included in the logistic regression analysis were excluded from the final model. AOR = Adjusted Odds Ratio. Ref = Reference category. AOR = Adjusted Odds Ratio. Ref = Reference category.

## Results

Of the 10,271 individuals included in the final analysis, 11.6% stated they never drank alcohol and 87.9% that they drank alcohol at least once every six months (0.5% did not respond). Those that drank were more likely to be female, older, attend a school in the least deprived regional quintile, white or mixed race, have more spending money available per week and/or be a member of a youth or sports group. Not surprisingly, they were much more likely to purchase alcohol (than non consumers) although the majority of those that drank (60.4%) stated that they did not buy their own alcohol.

Remaining analyses utilise only those individuals who drank alcohol (n = 9,029; 87.9% of the total sample) and examine predictive factors for bingeing, frequent drinking, drinking in public settings as well as composite risk variables of "any drinking risk" and "all drinking risks" (Table 2). Overall 38.0% of drinking respondents usually binged when drinking, approximately a quarter (24.4%) were frequent drinkers and half (49.8%) drank in public settings. Being aged 16 (c.v. 15) and having more money to spend per week were strongly related to increases in all three individual risk factors and to both composite risk variables ("any drinking risk" and "all drinking risks"; Table 2). Males were more likely to binge and drink frequently but were no more likely to drink in public settings than females. Deprivation was linked with drinking in public settings although frequent and binge drinking were not. Ethnicity, as well as being strongly related to whether youths drank (Table 1), also affected patterns of drinking. Thus, white and mixed race youths were more likely to binge than any other group and mixed race youths were also more likely to drink in public settings (Table 2).

Personal purchase of alcohol was individually related to all risky drinking behaviours. Thus, drinking in public settings more than doubled from 33.1% in those that do not buy their own alcohol to 75.3% amongst those that do. Similarly, levels of frequent drinking more than doubled (from 16.3% to 38.8%) and binge drinking rose from 32.1% to 50.4%. The combined effects of these increases meant that the proportion of individuals showing "all drinking risks" was four times higher in those that do buy alcohol themselves compared with those that do (Table 2).

Being a member of a youth club, group or team was linked to reductions in both frequency of drinking and drinking in public settings. However, it was linked to an increase in bingeing (Table 2). This mixed effect means that such membership had no significant relationship with whether individuals were in the composite "any drinking risk" category but was negatively related to being in "all drinking risks". Additional factors also strongly related to risky drinking behaviour were accessing alcohol through others. Here, getting alcohol from older siblings, friends (both under and over 18), and adults outside shops were all predictors of increased binge, frequent and public drinking (Table 2). Covertly taking alcohol from parents was also a predictor of frequent and public drinking. Importantly however, where parents bought alcohol for their children this had the opposite effect and was related to reductions in all individual risky drinking categories and in both "any drinking risk" and "all drinking risks" measures (Table 2).

Using logistic regression to adjust for confounding factors, Table 3 shows independent relationships between risky drinking behaviours and predictive variables. Here, binge drinking is associated with being male, Asian/Asian British, having more than £10 per week spending money and personal purchase of alcohol. Being bought alcohol by older siblings or friends were both also associated with bingeing. However, being given alcohol by parents remained a protective factor against bingeing (Table 3). Frequent drinking was also strongly associated with being male, having more spending money and personal purchase of alcohol. Further, as well as older siblings and friends (over 18) buying youths alcohol, getting adults outside shops to buy it and covertly taking it from parents were both predictors of frequent use. Only being a member of a youth club, group or team was protective against frequent use. In contrast to other risk factors, drinking in public places was higher in females (although not quite significantly so) and increased with deprivation up to quintile four (Table 3). It was also strongly related to both youths buying their own alcohol and levels of spending money available. Older siblings, friends and adults outside shops buying youths alcohol were all positively associated with increased public drinking. Again however, parents buying youths alcohol was protective against drinking in public settings (Table 3).

A parent buying their child alcohol was also protective against both composite factors of "any drinking risk" and "all drinking risks", while being a member of a youth group or team was protective against "all drinking risks" but had no significant relationship with "any drinking risk". However, youths buying their own alcohol, getting alcohol from adults outside shops, from friends, and having more money to spend were all related to having "any drinking risk" and "all drinking risks", while purchase of alcohol by older siblings was related to "any drinking risk" but not "all drinking risks" (Table 3).

## Discussion

Although this is one of the largest samples of youths to have been examined for drinking behaviour in England, it was not designed to be representative of the population. In addition, in order to ensure truthful responses the survey asked a minimum on personal demographics and consequently deprivation was assigned utilising school locations rather than more sensitive individual measures. Such factors limit both the analyses possible within the study and the opportunity to extrapolate data to wider populations. Further, as a cross-sectional study our results do not address cause and effect. However, the design of the study has allowed detailed examinations of relationships between risky alcohol consumption, sources of alcohol, places of consumption and other individual behavioural and demographic characteristics.

In fact, levels of at least any alcohol consumption by study participants (here, 87.9%) were broadly consistent with national data that show a prevalence of any alcohol consumption in the last twelve months of 91% (aged 15–16) [8]. However, rather than conducting detailed analysis of factors relating to any alcohol use, we have examined factors predicting risky drinking among youths that consume alcohol. Such analyses acknowledge that the vast majority of 15 to 16 year old youths in the UK drink alcohol (at least occasionally), that alcohol consumption in controlled environments (e.g. with family) may not always be detrimental to health [31,46] and that identifying factors relating specifically to alcohol misuse (c.v. consumption) are a critical part of developing harm reducing interventions.

Results identify binge, frequent and public drinking as all being strongly related to amounts of spending money youths have available. Such information offers at least three possible points for intervention. Firstly interventions could aim to reduce money available to young people or advise parents on improving their monitoring of what youths spend money on. Currently, teenagers (aged 12–16) in the UK typically receive almost £10 pocket money a week from parents [47] while over a third (37%) of 14 to 15 year olds work in a regular paid job during school term time [48]. However, public health considerations of how providing money to youths affects behaviour or of how parents may better control expenditure are poorly developed. Secondly, increasing the cost of alcohol may reduce access to alcohol and thus consumption. Unfortunately, in real terms affordability of alcohol has increased in the UK [49] and despite good evidence that increased costs can reduce alcohol consumption, particularly among young people[50], there appear to be no moves to increase alcohol taxes [51] or allow areas to manage alcohol prices locally through other means [52]. Finally, organised social and sporting activities which are attractive to young people should be made more widely available as an alternative to getting drunk. Currently there is little international evidence that providing specifically alcohol-free diversionary activities will reduce youth alcohol consumption [53]. However, our results (Table 3) at least support findings elsewhere that individuals involved in youth groups and sporting activities are less likely to exhibit risky drinking behaviours [54]. Some interventions have been developed in the UK and other countries with activities such as sports, theatre and crafts provided to encourage youths away from anti-social behaviour, including substance use [53,55,56]. However, such programmes currently affect only limited numbers of individuals in deprived areas [55,56] and our results (Table 3) suggest risky drinking behaviours occur across all deprivation quintiles. Consequently, rather than tightly focused interventions, further exploration is required of wider population approaches including school-based after-school activities and parental roles in encouraging participation in sports and other social ventures. Further, with membership of such groups potentially associated with greater binge drinking (Table 2), provision of better entertainment options for youths should incorporate safer drinking messages for participants.

Related to available income but independently associated with increased binge, frequent and public drinking is individuals buying their own alcohol. Those who purchase their own drink are nearly six times more likely to drink in public settings, three times more likely to drink frequently and twice as likely to usually binge (Table 3). However, all such alcohol purchasing should be controlled. It is illegal to sell alcohol to anyone under 18 in the UK and a range of voluntary identity schemes (e.g. Challenge 21) are available to ensure that those trying to buy alcohol are aged 18 or over [52]. Local enforcement bodies (e.g. Trading Standards, police) can punish and even force closure of establishments that persistently sell alcohol to those underage. However, despite evidence that such interventions are effective [57], at least historically such powers have been used infrequently. Even with increased enforcement in recent years, over a fifth of targeted test purchases still result in a positive sale to a minor (England and Wales)[58]. Moreover, in our sample approximately a third of the individuals had bought alcohol for themselves (39.6% of those that drink). With greater investment in enforcing underage sales restrictions these proportions could be radically reduced. Further, partnership working between alcohol retailers, health services and enforcement sectors could develop interventions to help those identified trying to buy alcohol underage.

Not all alcohol consumed by youths is purchased by them. In this study many individuals used older siblings, friends and even strangers outside alcohol retail outlets to obtain alcohol. Obtaining alcohol from older siblings was related to binge, frequent and public drinking and again consideration should be given to informing parents of additional risks faced by younger siblings from alcohol provided by older brothers and sisters [59]. Obtaining alcohol from adults outside shops was especially related to drinking in public settings. Not only does this allow youths to get drunk but it also requires young people to interact with adult strangers, potentially exposing children to the risk of sexual abuse. However, partnership working could allow adults who buy alcohol for underage youths to be identified and laws preventing such purchases enforced.

Finally, and in stark contrast to other forms of obtaining alcohol, youths whose parents buy them alcohol were less likely to binge and only half as likely to drink in public settings. Moreover, they were also significantly less likely to be in the most harmful group showing all three risk behaviours (Table 3). Such findings suggest that parental provision of alcohol, at least to youths who already consume, may reduce their immediate risk of hazardous and harmful consumption behaviours. These findings are consistent with other studies which identify drinking with parents as protective against higher alcohol consumption [60]. Further, although early initiation into alcohol use appears to be related to later problem drinking, those initiated in a family environment are much less likely to become problem drinkers than those initiated outside the family [61]. Models of family drinking in other (e.g. Mediterranean) countries, where alcohol is routinely consumed in the family environment but levels of binge drinking and anti-social behaviour associated with alcohol are lower than in the UK, are also at least consistent with a positive effects of parental alcohol provision. Such family consumption may help open up an early dialogue about alcohol between parents and children. Furthermore, it allows youths to experiment with alcohol in a family setting with positive parental role models rather than outside the family with pressure from peers to consume to excess. More research is needed on any positive effects of consuming alcohol in family environments. In the meantime however, it is essential that public health messages do not discourage parents from consuming modest amounts of alcohol with their children as such changes may actually increase drinking behaviours most damaging to youths' health.

## Conclusion

A combination of increases in binge, frequent and public drinking, mean alcohol is becoming one of the major threats to young people's health in many developed countries [6]. Such behaviour not only causes direct damage in the short term through accidents, violence and overdose but, in the long term, can lead to early onset of alcohol-related diseases such as liver cirrhosis [62]. Related anti-social behaviour disturbs and sometimes destroys communities and renders public spaces (e.g. parks and city centres) no-go areas for large numbers of individuals [63]. Here, we have shown strong links between the risky consumption of alcohol and factors such as expendable income and underage sales. We have also identified potentially protective factors against such consumption including parental provision of alcohol and engagement in other youth activities. In turn such intelligence points towards a series of interventions including: limiting and monitoring young people's funds; increasing costs of alcohol; providing and promoting participation in sporting and other social activities for youths; and identifying and closing all retailers selling to those underage. Such interventions are not expensive, complicated or difficult to implement. They require parents to examine children's expenditure and perhaps even moderately consume alcohol with them, educating them about its use and providing positive role models for responsible consumption. They also require the political willpower to eradicate any parts of the alcohol industry that provide alcohol to juniors. However, the effectiveness of such measures will still rely on youths having alternatives to alcohol and consequently local authorities and governments prioritising legitimate youth activities over more bars and nightclubs.

## Competing interests

The author(s) declare that they have no competing interests.

## Authors' contributions

MAB analysed the data and produced the manuscript. KH and MM assisted in production of the manuscript. KT, SH and EF assisted with data analysis. TA conducted the survey. DH edited the manuscript.
